# Clinical Therapeutic Strategy and Neuronal Mechanism Underlying Post-Traumatic Stress Disorder (PTSD)

**DOI:** 10.3390/ijms20153614

**Published:** 2019-07-24

**Authors:** Yasushi Yabuki, Kohji Fukunaga

**Affiliations:** Department of Pharmacology, Graduate School of Pharmaceutical Sciences, Tohoku University, Sendai 980-8578, Japan

**Keywords:** post-traumatic stress disorder, fatty acid binding protein 3, calcium/calmodulin-dependent protein kinase II, ramelteon

## Abstract

Post-traumatic stress disorder (PTSD) is characterized by an exaggerated response to contextual memory and impaired fear extinction, with or without mild cognitive impairment, learning deficits, and nightmares. PTSD is often developed by traumatic events, such as war, terrorist attack, natural calamities, etc. Clinical and animal studies suggest that aberrant susceptibility of emotion- and fear-related neurocircuits, including the amygdala, prefrontal cortex (PFC), and hippocampus may contribute to the development and retention of PTSD symptoms. Psychological and pharmacological therapy, such as cognitive behavioral therapy (CBT), and treatment with anti-depressive agents and/or antipsychotics significantly attenuate PTSD symptoms. However, more effective therapeutics are required for improvement of quality of life in PTSD patients. Previous studies have reported that ω3 long-chain polyunsaturated fatty acid (LCPUFA) supplements can suppress the development of PTSD symptoms. Fatty acid binding proteins (FABPs) are essential for LCPUFA intracellular trafficking. In this review, we have introduced *Fabp3* null mice as an animal model of PTSD with impaired fear extinction. Moreover, we have addressed the neuronal circuits and novel therapeutic strategies for PTSD symptoms.

## 1. Introduction

Post-traumatic stress disorder (PTSD), a profound public health issue, is most often induced by distressing events. During a lifetime, a majority of the population, 60.7% of men and 51.2% of women, may be exposed to traumatic events that have the potential to trigger the development of PTSD [1,2]. While previous studies have estimated the lifetime prevalence of PTSD in United State as 6.1–6.8%, and in Japan as 1.3% [3,4,5], 16.7% of the individuals exposed to the terrorist attack of September 11, 2001, experienced an onset of PTSD [6]. In addition, 5.7% of the survivors from the tsunami caused by The Great East Japan Earthquake on March 11 showed post-traumatic stress reactions [7]. Development and retention of PTSD are observed not only in the victims of disasters, but also in the rescue workers [8,9,10]. In the pathogenesis of PTSD, the fear memories are aberrantly consolidated, while the fear extinction fails to function [11]. Impaired fear memory consolidation and extinction triggers develop nightmares and flashbacks in patients with PTSD [2,11,12]. Patients with PTSD often show cognitive impairment, attention and/or learning deficits [13,14], which reduce the quality of life (QOL). According to clinical and animal studies, emotion- and fear-associated neuronal circuits are related to the development and retention of PTSD symptoms [11,15,16] and several therapeutics have been recommended [1,12,17,18]. However, mechanisms underlying enhanced susceptibility to impaired fear memory consolidation and extinction are not clear, and therefore, there are limited effective therapeutic approaches.

We, here in this review, discuss the neuronal circuits and a novel therapeutic strategy for PTSD symptoms. Moreover, we introduce a PTSD animal model to evaluate the efficacy of therapeutics in future investigations.

## 2. Neuronal Circuits in PTSD Symptoms

Animal studies suggest the crucial brain regions associated with PTSD symptoms as the prefrontal cortex (PFC), anterior cingulated cortex (ACC), amygdala, and the hippocampus, which are involved in the formation and retrieval of emotional and fear memory [19,20,21]. Destruction of the medial PFC (mPFC) by electrolytic lesion has shown to impair recall of fear extinction in rats [22]. The elevated neuronal firing rate in the infralimbic mPFC has been correlated with spontaneous recovery of freezing after the fear extinction, and short-term electric stimuli in infralimbic mPFC has shown to facilitate fear extinction memory in rats [19]. The infusion of γ-aminobutyric acid (GABA) receptor agonist, muscimol, into rat amygdala fails to acquire and consolidate fear memory [21]. Pharmacological lesions and inactivation in rat hippocampus have shown to block contextual fear memory formation [23,24]. Moreover, these brain regions are connected and regulated with each other [20,25,26]. In another study, the inactivation of basolateral amygdala (BLA) by infusion of muscimol decreased the neuronal activity in pyramidal neurons, as well as the fear conditioning tone response in rat prelimbic mPFC. Inversely, the inhibited ventral hippocampal activity enhanced the activity of interneurons and then promoted the tone response in rat prelimbic mPFC [25]. Optogenetic analyses have also indicated that the mouse BLA-mPFC synaptic inhibition impairs fear memory retention and facilitates fear extinction [27]. Therefore, neuronal connections between PFC, amygdala, and hippocampus may have an essential role in normal fear consolidation and extinction.

In addition, human studies also support the above discussed neuronal circuit in PTSD patients. The magnetic resonance imaging (MRI) studies have demonstrated reduced volumes of hippocampus and ACC in the brain of PTSD patients [28,29,30,31,32]. Functional MRI researches have also defined excessive activity in the amygdala, while hypofunction in both the mPFC and ACC in patients with PTSD when they are exposed to fear condition [33,34,35,36,37]. In contrast, several groups have reported that the activities of both the amygdala and ACC are higher in patients with PTSD than that in healthy subjects [38,39]; thus, the neuronal activity of ACC in PTSD is controversial. Brain blood flow is significantly decreased in the mPFC of patients with PTSD exposed to traumatic stimuli, whereas in non-PTSD subjects, it is elevated in ACC, indicating a negative relationship between mPFC and ACC [31,40]. In addition, synchronous neuronal activities between ACC and BLA are essential for a normal fear response [41,42]. Previous reports suggest that neuronal activities in the PFC, including mPFC and ACC, negatively function in BLA, thereby suppressing exacerbated fear response and anxiety [43,44,45]. According to clinical therapy reports, veterans with PTSD, who had been deployed to Iraq or Afghanistan, either showed improvement or normalization of the hyperactive amygdala and hypofunctional ACC on exposure therapy among individuals with PTSD during an affective Stroop response test [46,47]. Oxytocin treatment alleviates the impairment of the working memory, and concomitantly increases connectivity between the dorsolateral PFC and ACC in patients with PTSD [48]. Moreover, both pharmacological and psychological therapies have been reported to increase the volumes of the hippocampus and/or ACC, in association with the improvement of PTSD symptoms [49,50,51,52,53]. Taken together, these observations suggest that aberrant neuronal activities in PFC, ACC, amygdala, and hippocampus may be one of the reasons underlying development and retention of PTSD symptoms.

## 3. Clinical Therapeutics for Patients with PTSD

### 3.1. Psychological Therapy for PTSD Symptoms

According to National Institute for Clinical Excellence study (2005), Kar (2011) and Lee et al. (2016), psychological therapies, such as cognitive behavioral therapy (CBT) are recommended as the first-line therapy for PTSD symptoms [54,55,56]. CBTs, as represented by cognitive processing therapy, stress inoculation training, and written exposure therapy significantly improve PTSD symptoms [18,57,58,59,60,61]. Eye movement desensitization and reprocessing also attenuates PTSD symptoms [18,62,63,64]. The other types of psychological therapy highlight the effectiveness of imagery rehearsal therapy (IRT) and Seeking Safety (SS) in PTSD symptoms. However, supporting evidence for such therapies are inconclusive [18,65,66]. PTSD subjects undergoing CBTs have demonstrated better QOL before the treatment [61,67]. On the other hand, no differences in QOL have been observed between pre- and post-treatment with eye movement desensitization and reprocessing in patients with PTSD [18,62].

### 3.2. Pharmacological Therapy for PTSD Symptoms

Since imperative traumatic incidents, such as the terrorist attack and massive earthquake occur unexpectedly, and possess a strong potential to develop PTSD in the victims [6,7], pharmacological therapies are also crucial for the relief of PTSD symptoms. Clinical studies have demonstrated an improvement in PTSD by selective serotonin reuptake inhibitors (SSRIs), serotonin and noradrenaline reuptake inhibitor (SNRI), antipsychotics, and other drugs as listed in Table 1. SSRIs, such as fluoxetine, paroxetine, and sertraline significantly relieve PTSD symptoms and partially improve QOL in the PTSD subjects [64,68,69,70,71,72,73,74,75,76]. Treatment with SNRI, venlafaxine, also attenuates the PTSD symptoms and shows improvement in QOL [76,77]. The effects of another type of antidepressants on PTSD symptoms are relatively weaker than those of SSRI and SNRI [78,79,80,81]. Atypical antipsychotics risperidone, olanzapine, and aripiprazole have also been effective for PTSD symptoms (Table 1) [82,83,84,85,86,87]; however, evidence for their therapeutic potential is limited [18]. Importantly, risperidone and olanzapine relieve the PTSD symptoms that are resistant to SSRI treatment [84,86]. The efficacies of calcium channel blocker, alpha blocker, anticonvulsant, and synthetic cannabinoid on PTSD symptoms have also been reported (Table 1) [88,89,90,91,92,93,94]. However, only paroxetine and sertraline have been approved by the U.S. Food and Drug Administration (FDA) for PTSD therapy [18], and these pharmacological treatments are extremely limited, due to their side effects. Therefore, more safe and effective therapeutics for PTSD are required.

## 4. Animal Models of PTSD

PTSD animal models are essential to evaluate the efficacy of drugs and to reveal the neurochemical basis underlying the development and retention of PTSD. Stress-induced animal models have been proposed as PTSD models in various studies [110,111]. Rats subjected to electric stimulations from two different contexts show an exacerbated sensitization of the fear response [110]. Single prolonged stress (SPS) combined with restraint for 2 h, forced swimming for 20 min, and exposure to ether anesthesia has shown to induce hypersensitivity of glucocorticoid negative feedback with abnormalities in hypothalamic-pituitary-adrenal (HPA) axis in male rats, similar to that of patients with PTSD [111]. Studies have demonstrated that the rats subjected to SPS show sleep abnormalities, aberrant anxiety, enhanced contextual fear response, and impaired fear extinction [112,113,114,115]. Moreover, rats subjected to predator-based psychosocial stress (PPS; subjected to unstable housing conditions [changing housing pair] for 31 consecutive days, followed by 1 h of immobilization and exposure to a cat on day 1 and day 11) or predator scent stress (PSS; 10 min of inescapable exposure to well-soiled cat litter) exhibit PTSD-like anxiety behaviors and impaired fear extinction, which have been proposed as the symptoms in animal models of PTSD [116,117,118,119]. Additionally, many studies have focused on the process of fear extinction following contextual or cued fear conditioning in the rodents as a predominant symptom of PTSD [105,120]. Most of the drugs with the reported clinical efficacy have demonstrated anti-PTSD characteristics in the rodent models (Table 1) [95,96,97,98,99,100,101,102,103,104,106,107,108,109], suggesting that the efficacy of drugs was initially evaluated using the above animal models. However, whether the chronic stress-induced models reflect PTSD pathology is still questionable, due to the lack of reproducibility in different studies. 

To resolve these drawbacks, genetically modified animals have been useful in recent times for investigation of the neuronal mechanisms underlying the development and retention of PTSD. Several studies have indicated a relationship between PTSD symptoms and the serotonergic system. Serotonin 1A (5-HT_1A_) receptor knockout (KO) mice exhibit elevated anxiety-like behavior and fear retrieval [121,122,123]. On the other hand, lack of 5-HT_1A_ receptor does not affect the process of fear extinction in mice [123], suggesting that 5-HT_1A_ receptor may be associated with fear memory retrieval and consolidation. Additionally, deletion of the gene coding for 5-HT transporter in mice impairs fear extinction recall and enhances PTSD-like behaviors following exposure to predator odor [124,125]. Deficiency of pheochromocytoma 12 ETS (E26 transformation-specific) factor (Pet-1) has shown to reduce the mRNA levels of tryptophan hydroxylase 2 (TPH2) and 5-HT_1A_ receptor, as well as the immunoreactivities of TPH2 and 5-HT in dorsal raphe [126]. Following the reduction of the serotonergic system in the mouse brain [126], Pet-1 KO mice show increased anxiety, aggressive behaviors, and elevated fear response [127,128]. Pet-1 KO mice also display delayed fear extinction learning, but it does not affect the process [128]. Moreover, fMRI analyses have revealed that the threat-induced amygdala hyperactivation is associated with human Pet-1 variant [128]. Thus, hypofunction of serotonergic systems may reflect one of the aspects of the PTSD symptoms. Additionally, other genetic models have been proposed. The brain-derived neurotrophic factor, Met allele, has been associated with PTSD and impairment of fear extinction learning in mice [129,130]. Conditional KO of the corticotrophin-releasing hormone receptor type-1 gene in the limbic forebrain of mouse decreases remote associative and non-associative fear memory [131]. Mice with neuropeptide Y gene KO show potentiated acquisition of conditioned fear memory and impaired fear extinction [132]. Moreover, mice with overexpressed adult cholecystokinin receptor-2 display PTSD-like behavioral deficits when subjected to traumatic stimuli (foot-shocks) during puberty [133], suggesting that the interaction of genetic factor with the early environmental condition have an impact on the post-pubertal behavioral phenotype. These observations suggest that various genes are involved in altering the associated neuronal networks in PTSD; however, the molecular mechanism of fear extinction processing remains unclear. 

## 5. Role of Long-Chain Polyunsaturated Fatty Acids (LCPUFAs) and Fatty Acid Binding Proteins (FABPs) in the Brain

In the brain, LCPUFAs are an essential component of membrane phospholipids and important for brain development [134,135]. Aberrant metabolism of LCPUFAs has been reported in various psychiatric diseases. For example, levels of ω3 and ω6 LCPUFAs in the membrane of red blood cells are decreased in the schizophrenia subjects, while ω3 LCPUFA levels are reduced in the plasma of the autistic subjects [136,137,138]. Likewise, the concentration of ω3 LCPUFA is significantly decreased in the erythrocytes and plasma of PTSD patients [139]. Consistent with the observation, ω3 LCPUFA supplementation has shown to prevent the development of PTSD and also reduce the PTSD symptoms after accidental injuries, including the Great East Japan Earthquake [140,141,142]. Studies have also demonstrated that the effect of ω3 LCPUFAs on the PTSD symptoms could be due to the elevation in the hippocampal neurogenesis [140,141], since the hippocampus-dependent fear memory is closely associated with the activity of neurogenesis in mouse hippocampal dentate gyrus (DG) [143]. Indeed, ω3 LCPUFA administration has shown to facilitate mouse and rat hippocampal neurogenesis [144,145]. Taken together, disturbances in the LCPUFA supplementation and metabolism may be associated with the PTSD symptoms.

FABPs, low molecular weight (14–15 kDa) proteins with twelve subtypes in most mammals, have a key role in the intracellular uptake, transport, and metabolism of LCPUFAs [146,147]. In the mouse and human brain, three types of FABPs, FABP3, FABP5, and FABP7 are primarily expressed [148]. While FABP5 and FABP7 are localized in the glial and neuronal stem/progenitor cells, FABP3 is expressed in the mature neuronal cells [148,149]. Lack of *Fabp5* and/or *Fabp7* reduces the proliferation of neural stem cells in the mouse hippocampal DG [149], and *Fabp7* null mice exhibit impaired emotional behaviors, including aberrant fear response, sensory motor dysfunction, and sleep disturbance [150,151]. We previously have demonstrated that FABP3 interacts with the dopamine D2 receptor long isoform (D2LR), and that deficiency of *Fabp3* reduces methamphetamine-induced sensitization and increases haloperidol-induced catalepsy, due to the dysfunction of dopamine D2 receptors [152,153]. Indeed, *Fabp3* null mice are resistant to dopaminergic toxicity-induced parkinsonism [154]. Moreover, *Fabp3*, *Fabp5,* and *Fabp7* gene variants have been identified in patients with schizophrenia and autism spectrum disorder [155]. These observations suggest that dysfunction of FABPs in the brain is associated with the development of a psychiatric disorder.

## 6. Impaired Fear Extinction Process in *Fabp3* Null Mice

**Figure 1:**
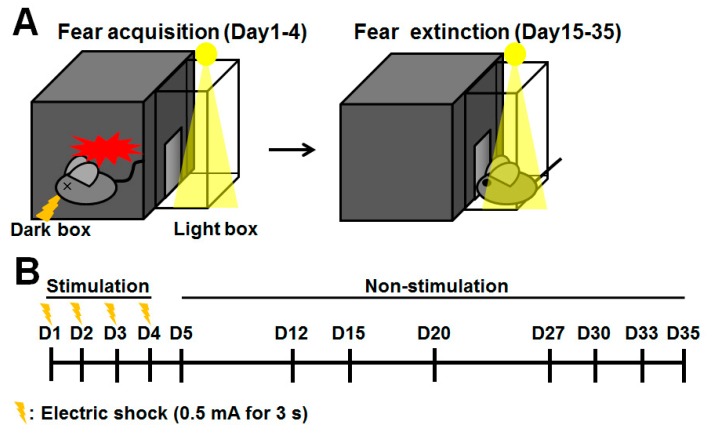
Experimental apparatus and design for evaluation of fear extinction. (**A**) Schematic diagram of contextual fear memory in passive avoidance task to evaluate fear extinction in the mouse. (**B**) Experimental schedule for assessment of fear extinction. Mice received an electric shock (0.5 mA for 3 s) once a day for four consecutive days (fear acquisition). After that, the mouse was exposed to the light box, and step-through latency was measured without an electric shock (fear extinction). D: Day.

**Figure 2:**
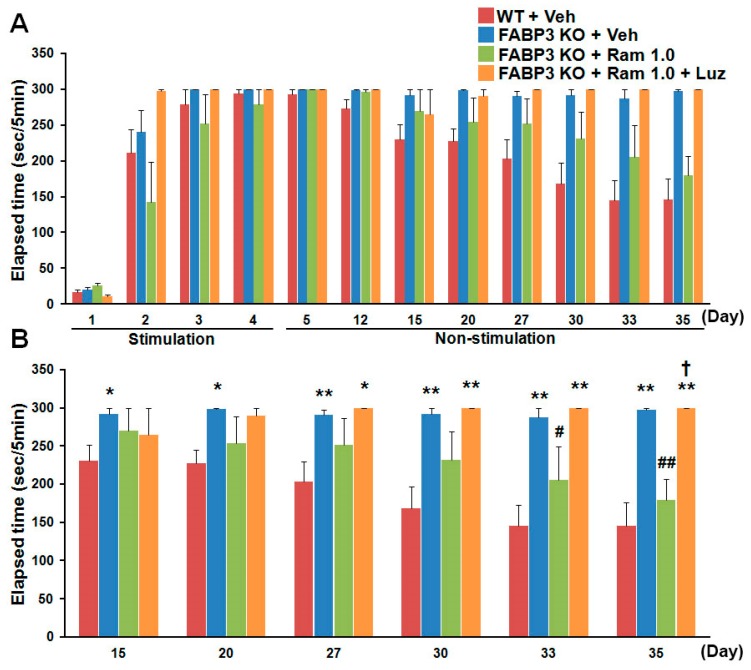
Impaired fear extinction processing in *Fabp3* null mice. (**A**) Overview of fear responses as elapsed time during the acquisition (Day 1 to 4) and extinction phase (Day 5 to 35) (*n* = 4–11 per group). (**B**) Ramelteon (1.0 mg/kg, p.o.) repaired the impaired fear extinction process in *Fabp3* null mice from Day 15 to 35, an effect prevented by luzindole (2.5 mg/kg, i.p.) treatment (*n* = 4–11 per group). Error bars represent SEM. * *p* < 0.05 vs. vehicle-treated wild-type (WT) mice; ** *p* < 0.01 vs. vehicle-treated WT mice; # *p* < 0.05 vs. vehicle-treated *Fabp3* null mice; ## *p* < 0.01 vs. vehicle-treated *Fabp3* null mice; † *p* < 0.05 vs. ramelteon (1.0 mg/kg, p.o.)-treated *Fabp3* null mice. Luz, luzindole (2.5 mg/kg, i.p.) treatment; Ram 1.0, ramelteon (1.0 mg/kg, p.o.) treatment; veh, vehicle treatment. Modified data derived from Reference [156].

**Figure 3:**
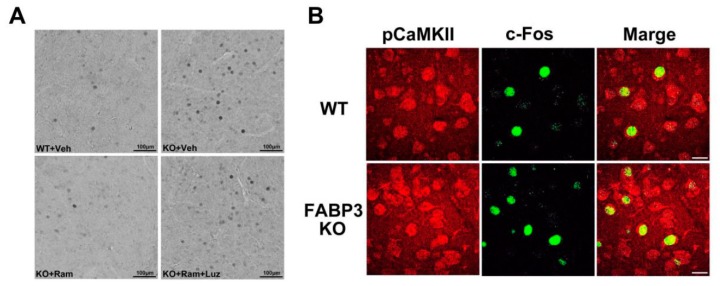
Aberrant c-Fos expression and CaMKII autophosphorylation levels in the basolateral amygdala (BLA) in *Fabp3* null mice. (**A**) Representative images of BLA in each group of mice one hour after the extinction test on Day 35. The effect of ramelteon (1.0 mg/kg, p.o.) on the elevated level of c-Fos was antagonized by luzindole (2.5 mg/kg, i.p.) treatment in *Fabp3* null mice. Scale bars: 100 µm. (**B**) Representative images of BLA in WT and *Fabp3* null mice stained by phosphorylated CaMKII (red) and c-Fos (green). Increased c-Fos expression levels were observed in elevated autophosphorylation CaMKII-positive cells in BLA of *Fabp3* null mice. Modified data derived from Reference [156].

To assess the fear process of extinction acquisition and extinction, a mouse is placed in a box with dark and light compartments, shown in Figure 1, and the step-through latency is recorded up to 300 sec with or without electric shock (Day 1–4; Figure 1). No differences were observed during fear acquisition (Day 1–4) and retrieval (Day 5) between wild-type (WT) and *Fabp3* null mice (Figure 2). WT mice showed normal fear extinction process from Day 12 to Day 35 gradually; however, *Fabp3* null mice failed, suggesting that *Fabp3* deficiency impairs fear extinction processing (Figure 2) [156]. *Fabp3* null mice also exhibited cognitive impairment, increased daytime locomotor activity, which may reflect sleep disturbance, and anxiety behaviors [156,157], suggesting that *Fabp3* null mice may display PTSD-like behaviors. Consistent with clinical investigations, the level of c-Fos, as an indicator of neuronal activity, was markedly elevated in the BLA of *Fabp3* null mice one hour after the exposure of fear on Day 35 (Figure 3a,b) [156]. *N*-methyl-*d*-aspartate (NMDA) receptor signaling plays an essential role in fear response and c-Fos expression [158,159,160], and one of its major downstream targets calcium/calmodulin-dependent protein kinase II (CaMKII) phosphorylation levels are significantly reduced in the ACC and conversely elevated in the BLA of *Fabp3* null mice as compared to WT mice [156], indicating hypofunction and hyperactivation in the *Fabp3* null ACC and BLA, respectively. FABP3 is highly localized in the parvalbumin-positive GABAnergic neurons in mouse ACC, and the GABAnergic neuronal activities are elevated in the ACC of *Fabp3* null mice, thereby attenuating glutamatergic neurotransmission [157]. Since the neuronal activity of ACC negatively regulates BLA neuronal activity and thus, suppresses overactivation of BLA neuronal circuit [43,44,45]. We, therefore, speculate that the hypofunction in ACC fails to suppress aberrant BLA neuronal activation during fear conditioning, thereby, impairing the process of fear extinction in *Fabp3* null mice. These changes in the brain activities of *Fabp3* null mice are likely to be similar to that observed in subjects with PTSD [33,34,35,36,37,38,39,46,47], and moreover, this is first report to demonstrate the relationship between FABP and PTSD, suggesting that *Fabp3* null mice are useful genetic models to study the pathophysiology, and assess the effect of novel candidate drugs on PTSD symptom.

## 7. Potential Efficacy of Melatonin Receptor Agonist for PTSD Symptom

**Figure 4:**
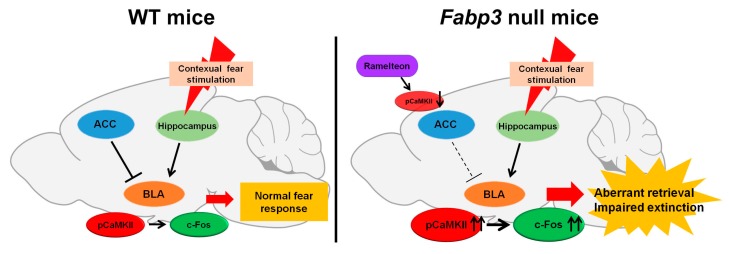
The hypothesis of neuronal circuits in the fear extinction in WT and *Fabp3* null mice. (Left) Contextual fear memory is acquired in the hippocampus and then consolidated and retrieved in the BLA. The ACC negatively regulates neuronal activity in BLA, resulting in suppression of exacerbated fear retrieval and facilitation of fear extinction in WT mice. (Right) Hypofunction in the ACC fails to inhibit aberrant neuronal activity in the BLA, leading to the development of aberrant fear retrieval and impaired fear extinction in *Fabp3* null mice. Ramelteon improves decreased neuronal activity in the ACC through stimulation of the melatonin receptors. Therefore, ramelteon improves the impaired fear extinction by suppressing hyperactivation of BLA in *Fabp3* null mice (Right). Modified data derived from Reference [170].

Melatonin, a pineal hormone synthesized from serotonin, mediates circadian rhythms, sleep, mood, and cognition [161,162], and Huang et al. in their study observed that melatonin treatment facilitates fear extinction in naïve rats [163]. The melatonin receptor selective agonist, ramelteon, shows a 3- to 7-fold higher affinity and 5.5- to 9.6-fold greater potency for recombinant monkey and human MT1/MT2 receptors than that of melatonin and has been approved for insomnia in clinical therapy [164]. Therefore, we evaluated the effect of ramelteon on impaired fear extinction in *Fabp3* null mice. Administration of ramelteon (1.0 mg/kg, p.o.) significantly relieved not only PTSD-like symptom, including impaired fear extinction, but also pathological changes in *Fabp3* null mice (Figure 2, Figure 3a). Melatonin receptor antagonist, luzindole (2.5 mg/kg, i.p.) treatment prevents the effect of ramelteon in *Fabp3* null mice (Figure 2, Figure 3a), suggesting that improvement by ramelteon may be due to activating the melatonin receptors. Importantly, melatonin MT1 and MT2 receptors are highly expressed in rodent PFC, including ACC, and are almost absent in the amygdala [165]. Since treatment with melatonin enhances the reduced CaMKII autophosphorylation levels in the hippocampal CA1 region and then improves the cognitive impairment in an animal model of autism [166], we suggest that ramelteon initially improves the decreased CaMKII activity (neuronal activity) in the ACC and in turn suppresses the elevated neuronal and CaMKII activities in the BLA, thereby, reversing the impaired fear extinction in *Fabp3* null mice (Figure 4). 

The previous report indicates that stimulation of Gi-coupled receptor triggers the Gβγ-phospholipase Cβ (PLCβ) pathway and then elevates intracellular calcium levels by activating the inositol 1,4,5-trisphosphate receptors [167], suggesting one of the mechanisms underlying CaMKII activation by stimulation of melatonin receptors. In an ex vivo experiment, melatonin incubation in the brain slices enhanced the dendrite length, thickness, and complexity in the rat hippocampal neurons via CaMKII activation [168]. Glutamate (10 μM) application failed to activate calcium signaling, including CaMKII in mouse cortical primary neurons on DIV 21, due to spontaneous firing; however, it significantly enhanced CaMKII autophosphorylation levels under melatonin (5 μM) treatment [169]. Since levels of melatonin in the rat brain fluctuate with the circadian rhythm [170], CaMKII activation by glutamate application with melatonin treatment may reset the circadian rhythm in the suprachiasmatic nucleus in mice [170]. Additionally, NMDA receptor partial agonist, D-cycloserine, has demonstrated to facilitate fear extinction in animal models and patients with PTSD [171,172,173]. Taken together, hypofunction in ACC may fail to attenuate neuronal hyperactivity in BLA after contextual fear retrieval and in turn may impair fear extinction in *Fabp3* null mice. Furthermore, ramelteon can antagonize the PTSD-like behaviors by MT receptor stimulation, and, hence, suppress the hyperactivation of BLA in *Fabp3* null mice (Figure 4). Although the mechanism of action of ramelteon on CaMKII activation through the stimulation of Gi protein-coupled melatonin receptors is not clear, recent work on the protective functions of melatonin, especially in cognitive impairment implies the involvement of other kinases [174].

Although the clinical trial of patients experiencing PTSD with melatonin or ramelteon has not been conducted yet, reduced melatonin levels in the first 48 h after exposure to traumatic stress may be associated with a higher risk for PTSD [175]. Since the melatonin system plays an important role in sleep, cognitive function, pain, neuroimmunomodulation, stress response (HPA axis), circadian rhythm, and oxidative stress, all of which are affected in case of PTSD, upregulation of the melatonergic system could provide a potentially promising treatment strategy in the management of PTSD symptoms [176,177]. Here, we demonstrate the effect of ramelteon on PTSD-like behaviors in *Fabp3* null mice. As ramelteon has been approved for treating sleep disturbance, the schedule of administration will be considered. In conclusion, we propose and hope that ramelteon is repurposed as a novel therapeutic for treating PTSD in the near future.

## 8. Conclusions

In the present article, we have reviewed the predicted neuronal circuits and evidenced therapeutics in PTSD symptoms according to clinical and basic animal studies. Additionally, we introduce and discuss *Fabp3* null mice as a useful animal model of PTSD to investigate the neurochemical basis of fear extinction processing. Similar to clinical observations, impaired neuronal activities in the ACC and BLA were found to be associated with PTSD-like behaviors in *Fabp3* null mice, suggesting the potential role of FABP3 in PTSD. In another study, decreased levels of stress-related intracellular molecules, such as serum/glucocorticoid regulated kinase 1 and FK506 binding protein 5 were observed in the PFC of PTSD subject’s postmortem, and also have been associated with fear response in rodents [44]. Melatonin receptor activation reduces cyclin-dependent kinase 5 (Cdk5) expression [178], and this increase in Cdk5 expression attenuates fear memory retrieval [179]; therefore, the relationship between the dopamine D2 receptors and PTSD-like behaviors in *Fabp3* null mice can be explored. Further studies are necessary to establish the mechanisms underlying the impaired fear extinction in *Fabp3* null mice. On the other hand, melatonin receptor agonist, ramelteon, antagonized the PTSD-like behaviors in *Fabp3* null mice, and therefore, we suggest that melatonin receptors may be a novel therapeutic target and ramelteon can be an effective drug candidate for the PTSD symptoms. As ramelteon has been approved for insomnia, preclinical studies and clinical trials will be helpful in the establishment of ramelteon as a PTSD therapy in the near future.

## Figures and Tables

**Table 1:** Effects of clinical drugs in patients with post-traumatic stress disorder (PTSD) and animal models of PTSD.

Drug Treatment	Effects on PTSD Symptoms	Animal Models
**SSRI**		
Fluoxetine	Improvement [64,68,69], Not effective [70]	Facilitation of fear extinction [95,96]
Paroxetine	Improvement [71,72]	Prevention of PTSD symptoms reactivation [97]
Sertraline	Improvement [73,74,75]	No effect on fear extinction [98]
**SNRI**		
Venlafaxine	Improvement [76,77]	Facilitation of fear extinction [99]
**Anti-depressant**		
Mirtazapine	Improvement [80], Not effective [18]	Relief of fear response [100,101]
Bupropion	Not effective [81]	Relief of fear response [100]
**Antipsychotics**		
Risperidone	Improvement [82,83,84]	Facilitation of fear extinction [102]
Olanzapine	Improvement [85,86]	Relief of fear response [102], deficits of fear extinction [103]
Aripiprazole	Improvement [87]	Facilitation of fear extinction [104]
Sulpiride	No data	Facilitation of fear extinction [105]
**Other drugs**		
Gabapentin (Calcium blocker)	Improvement [88]	Relief of anxiety response [106]
Prazosin (Alpha blocker)	Improvement [89,90], Not effective [91]	Relief of fear response [107], facilitation of fear extinction [108]
Topiramate (Anticonvulsant)	Improvement [92,93]	Facilitation of fear extinction [109]
Nabilone (Cannabinoid)	Improvement [94]	No data

## Abbreviations

ACC	anterior cingulated cortex
BLA	basolateral amygdala
CaMKII	calcium/calmodulin-dependent protein kinase II
CBT	cognitive behavioral therapy
Cdk5	cyclin-dependent kinase 5
DG	dentate gyrus
EMDR	eye movement desensitization and reprocessing
FABP	fatty acid binding protein
fMRI	functional magnetic resonance imaging
GABA	γ-aminobutyric acid
HPA	hypothalamo-pituitary-adrenal
5-HT	serotonin
IRT	imagery rehearsal therapy
LCPUFA	long-chain polyunsaturated fatty acid
MRI	magnetic resonance imaging
NMDA	*N*-methyl-*d*-aspartate
PFC	prefrontal cortex
PTSD	Post-traumatic stress disorder
QOL	quality of life
SNRI	serotonin and noradrenaline reuptake inhibitor
SPS	Single prolonged stress
SSRI	selective serotonin reuptake inhibitor
WT	wild-type
